# Antibiotic stewardship program in Pakistan: a multicenter qualitative study exploring medical doctors’ knowledge, perception and practices

**DOI:** 10.1186/s12879-021-06043-5

**Published:** 2021-04-21

**Authors:** Muhammad Atif, Beenish Ihsan, Iram Malik, Nafees Ahmad, Zikria Saleem, Azka Sehar, Zaheer-ud-Din Babar

**Affiliations:** 1 Department of Pharmacy Practice, Faculty of Pharmacy, The Islamia University of Bahawalpur, Bahawalpur, Pakistan; 2 Department of Pharmacy Practice, Faculty of Pharmacy and Health Sciences, University of Balochistan, Quetta, Pakistan; 3 Department of Pharmacy, University of Lahore, Lahore, Pakistan; 4 Department of Pharmacy, University of Huddersfield, Huddersfield, UK

**Keywords:** Antibiotic stewardship, Antibiotic resistance, Rational use, One health

## Abstract

**Background:**

The emerging threat of antibiotic resistance is growing exponentially and antibiotic stewardship programs are cornerstone to fight against this global threat. The study aimed to explore the knowledge, perspectives and practices of physicians regarding various aspects of antibiotic stewardship program including antibiotic stewardship activities, rational use of antibiotics, antibiotic resistance, prescribing practices and factors associated with these practices.

**Methods:**

In this qualitative study, a total of 17 semi-structured, in-depth interviews with doctors of three tertiary care public sector hospitals in Bahawalpur and Rahim Yar Khan were conducted. The convenient sampling method was adopted to collect the data and the saturation point criterion was applied to determine the sample size. Thematic analysis approach was used to draw conclusions from the data.

**Results:**

The analysis of data yielded five themes, 12 subthemes and 26 categories. The themes included, (i) perception about antibiotic use and antibiotic stewardship, (ii) antibiotic prescription practices, (iii) antibiotic resistance, (iv) limited strategies adopted by hospital administration to ensure quality and safe distribution of antibiotics, (v) implementation of antibiotic stewardship program: barriers, suggestion and future benefits. Doctors had misconceptions about the rational use of antibiotics. The perception regarding antibiotic stewardship programs was poor. Moreover, very few activities related to ASP existed. The participants gave many suggestions for successful implementation of ASP in order to reduce the burden of antibiotic resistance, including development of guidelines for the use of antibiotics, strict legislation regarding use of antibiotics, active participation of healthcare professionals and awareness program among general public about the use of antibiotics.

**Conclusion:**

This study concluded that poor knowledge of doctors regarding ASP, non-existence of antibiogram of hospital and lack of rules for the safe use of antibiotics were the main driving factors associated with irrational antibiotic prescription practices and development of AR.

**Supplementary Information:**

The online version contains supplementary material available at 10.1186/s12879-021-06043-5.

## Background

Antibiotics are lifesaving drugs, and more than half of the hospitalized patients receive antibiotics [1, 2]. According to a study, global consumption of antibiotics increased by 65% between 2000 and 2015, and this increase was driven by increased consumption in low- and middle-income countries (LMICs). During the same time frame (i.e., 2000 to 2015), the total amount of antibiotics consumed in high income countries (HICs) increased marginally by 6% (i.e., 9.7 to 10.3 billion defined daily dose (DDD)), whereas in LMICs, antibiotic consumption increased 114% (i.e., 11.4 to 24.5 billion DDDs) [3]. In 2015, the LMICs with the highest consumption of antibiotics were India, China and Pakistan [3, 4]. Alarmingly, between 2000 and 2015, antibiotic consumption increased 65% (i.e., 0.8 to 1.3 billion DDDs) in Pakistan [3].

Over the past few years, unnecessarily overuse of antibiotics has led to the emergence of multidrug resistant (MDR) bacterial strains making them ineffective [5–7]. According to the Centers for Disease Control and Prevention (CDC), MDR bacteria cost 23,000 lives annually [8]. Alarmingly, antibiotic resistance (AR) will cause 10 million deaths by 2050, if timely measures are not taken [9]. Besides the development of MDR bacterial strains, inappropriate use of antibiotics negatively influences patients’ lives by increasing the length of hospitalization and out-of-pocket expenses, and decreasing quality of life of patients. Moreover, it leads to the financial burden on a healthcare systems [10–16]. Antimicrobial Stewardship involves adopting systematic measures to optimize antimicrobial use, decrease unnecessary antimicrobial exposure and to decrease the emergence and spread of resistance. Many developed countries, such as, Colombia, the United States (US), Australia, South Africa, and the United Kingdom (UK), have developed and successfully implemented different approaches to halt the spread of AR [17]. Regardless of having a high prevalence of AR, LMICs struggle to adhere to antibiotic stewardship programs [18] owing to limited resources [19].

The main factors increasing rates of MDR infections in Pakistan include the unwarranted use of broad spectrum antibiotics as well as insufficient infection prevention and control (IPC) and antibiotic stewardship polices [15, 20–22]. Consequently, a surge in AR has been seen over the past years, which makes drug therapy complex for infectious diseases like typhoid, tuberculosis, etc. [20, 23]. In 2016, the Medical Microbiology & Infectious Disease Society of Pakistan adopted the guidelines provided by the World Health Organization (WHO) Global Action Plan (GAP) to combat antimicrobial resistance, and started antibiotic stewardship program (ASP) in hospital settings [24–26]. The aim of this GAP was to halt the progression of antimicrobial resistance by initiating interventions at various levels of healthcare such as institutions, prescribers, policy makers and patients [27]. Moreover, this program also aimed to improve the clinical outcomes among the patients on antimicrobial therapy [27]. A few earlier studies from Pakistan showed that despite the efforts to implement ASP in Pakistani hospitals, the antibiotic use practices were inappropriate and the physicians were least aware about this program [21, 28–31]. The most common antibiotic stewardship activities currently practiced in Pakistani hospitals include clinical pharmacy services, the use of antimicrobial prescribing guidelines, consultation services, use of drug and therapeutics committees to approve antimicrobial prescribing, regular audit by doctors on antimicrobial prescribing and use of a restricted formulary for antimicrobial. Nevertheless, the aforementioned studies were conducted in Lahore which is the capital of the Punjab province and located in the northern part of the Punjab province of Pakistan, while similar studies from the southern Punjab are scarce. Owing to relatively better healthcare infrastructure in the capital city of Punjab, the findings of the above-mentioned studies cannot be extrapolated to the whole of Punjab. Moreover, perceptions of physicians about ASP, AR and antibiotic could vary from institution to institution. Therefore, a multicenter qualitative study was conducted with the aim investigate ASP in the public sector hospitals of the southern Punjab province of Pakistan. The study emphasized on knowledge, perspectives and practices of doctors regarding various aspects of ASP including the rational use of antibiotics, antibiotic resistance, prescribing practices and factors associated with these practices. The findings of this study will help to understand the perspective of doctors about antibiotic prescription practices and their perception about ASP. This will also help the policymakers understand the barriers associated with the successful implementation of this program in Pakistani hospitals.

## Method

### Study setting

In Pakistan, healthcare in the country is regulated by the Ministry of National Health Services Regulation and Coordination and the healthcare delivery system is comprised of primary, secondary and tertiary care [32]. Primary care is provided by the basic health units and the rural health centers and lady health workers. The district headquarter hospitals and the tehsil headquarter hospitals provide secondary care [32]. Tertiary care is provided by the tertiary hospitals, mainly situated in highly populated and developed parts of the country [32]. Overall, the Pakistani health sector is confronted with an imbalance in the number and deployment of healthcare workforce, and inadequate allotment of resources across different tiers of healthcare [33]. There are 132,227 hospital beds, 1279 hospitals, 5671 dispensaries, 5527 basic health units, and 747 maternity and child health centers in the country [34]. To serve the healthcare needs of a population of more than 207 million citizens, there are 233,261 doctors, 24,930 dentists, 112,123 nurses and 20,565 lady health workers in the country [34].

This study was conducted in two cities of the Punjab province of Pakistan i.e., Bahawalpur and Rahim Yar Khan. The Bahawal Victoria Hospital (BVH), Bahawalpur (1600 plus bedded facility) and the Civil Hospital, Bahawalpur (400 bedded facility) are two tertiary care hospitals located in the district of Bahawalpur [13, 35], and the Sheikh Zayed Hospital, Rahim Yar Khan (1700 bedded facility) is the only tertiary care hospital located in the district of Rahim Yar Khan. The provision of sub-optimal ASPs in these hospitals are limited to the use of antimicrobial prescribing guidelines, delivery of infectious diseases consultation services, use of drug and therapeutics committees to approve antimicrobial prescribing, regular audit by prescribers and use of a restricted formulary for antimicrobial [12]. Medical doctors working in these three hospitals were invited to participate in the study.

### Study design and study participants

A qualitative study design [36] was adopted to evaluate the knowledge and perception of doctors about ASP and related activities in their hospitals. The doctors who consented to participate in the study were face-to-face interviewed using semi structured interview guide. Doctors having working experience of ≥2 years were included in this study. The interviews were conducted from the doctors having different specializations to understand the views of doctors from different specialties.

### Interview schema development

The interview schema was developed after comprehensive literature review to answer the research questions (Additional file 1) [36, 37]. The schema was developed keeping in mind the inductive approach for data analysis because the creation of preliminary codebook to guide deductive analysis [38] was not possible due to unavailability of data on our research question, especially from Southern Punjab (study setting). Therefore we included open-ended questions for in-depth exploration of various aspects of the ASP. Semi structured interview schema was pilot tested in two doctors to ensure its validity, and uniformity of the content. It helped in better understanding of study’s instrument. The results of pilot interviews were not included in the final analysis. Final pilot tested interview guide included 12 questions with further sub questions to evaluate the knowledge of doctors about ASP and its related activities in hospitals, reasons of unnecessary use of antibiotics and their perspective on situation of resistance.

### Data collection

The data were collected in July and September, 2019. The participants were selected by two step sampling processes. In the first step, purposive sampling technique was adopted with the aim to ensure information richness and representation of the population [39]. Resultantly, a list of 54 knowledgeable experts having work experience of ≥2 years and working in different specialties (where antibiotics are frequently prescribed e.g., kidney ward, emergency department, paediatrics etc.) was generated. Thereafter, considering the busy schedules of both the physicians and the research team, we proceeded with the convenient sampling. Participants were approached between 8 am to 2 pm in hospitals and those who consented to participate were interviewed at a time and place that was agreeable to both parties. BI and AS were involved in data collection. They had prior training in qualitative studies before data collection. All interviews were audio recorded and conducted in Urdu (national language of Pakistan). The sample size was determined by applying the saturation point criteria [40, 41]. Two additional interviews were conducted to confirm the saturation in emergent themes.

### Data analysis

All audio-taped interviews were transcribed verbatim. The written transcripts were translated into English and read several times by BI and IM to familiarize and deeply understand the data. Forward-backward translation method was applied on some of the transcripts to ensure the accuracy of translation [42]. The data were analyzed using thematic content analysis. Standard method of the inductive thematic analysis was followed [36, 43]. First of all, all the relevant words and sentences were highlighted and codes were assigned to them. After coding the lines from the interviews, the data were organized into relevant categories. The categories were then reduced to draw final themes and subthemes [44, 45]. Themes were decided to answer the research questions posed. Suitable quotations were added for the understanding of themes and subthemes. MA led the data analysis process and gave the final verdict in case there was any disagreement among the authors. We followed Consolidated Criteria for Reporting Qualitative Studies (COREQ) during the study process [46].

## Results

### Participant’s characteristics

A total of 17 doctors were interviewed. Though, the saturation in emergent codes and themes was achieved after 15 interviews, but two additional interviews were conducted to confirm that the saturation was achieved. Four doctors did not agree to participate in the interviews during the convenient sampling stage due to assorted reasons, such as lack of interest in the study, fear of authorities and busy work schedule (response rate = 80.9%). The duration of interviews ranged from 20 to 30 min with an average duration of 25.5 min (SD = 3.83). The participants had minimum experience of two years and maximum experience of 10 years. Eleven participants were male and six were female. Characteristics of the participants are given in Table 1.

**Table 1:** Characteristics of the respondents

Respondent	Sex	Specialization	Interview duration
Doctor 1	Female	Pulmonology	32
Doctor 2	Male	Medicine	29
Doctor 3	Male	Pulmonology	22
Doctor 4	Female	Pediatrics	27
Doctor 5	Male	Pediatrics	22
Doctor 6	Female	Gynecology	23
Doctor 7	Male	Cardiac surgery	30
Doctor 8	Male	Nephrology	28
Doctor 9	Male	Nephrology	27
Doctor 10	Male	Pediatrics surgery	25
Doctor 11	Female	Burn unit	25
Doctor 12	Male	Nephrology	20
Doctor 13	Male	Cardiac surgery	30
Doctor 14	Male	Pediatrics	24
Doctor 15	Female	Pediatrics	30
Doctor 16	Female	Pediatrics	20
Doctor 17	Male	Pediatrics	20
**Mean duration**			**25.5 min**

The thematic analysis of the data yielded five themes and 12 subthemes. The themes included: perception about antibiotic use and ASP, antibiotic prescription practices, AR, limited strategies adopted by hospital administration to ensure quality and safe distribution of antibiotics, and implementation of ASP: barriers, suggestion and future benefits.

### Theme 1: perception about antibiotic use and antibiotic stewardship

To understand the familiarity of doctors about the terms related to antibiotic use, they were asked about how they perceive the term rational use. Most of the study participants had inadequate knowledge about rational use of antibiotics (12 out of 17 respondents) as they had misconception that use of broad spectrum antibiotics is called empirical therapy and that it was rational use. All respondents (17 out of 17 respondents) agreed that irrational use of antibiotics was a problem in Pakistan. ASP was the term that most of the doctors were not familiar with. Doctors who were familiar with the term were those who had work experience at large private sector hospitals in Karachi and Lahore (3 out of 17 respondents) (Table 2).

**Table 2:** Perception about antibiotic use and antibiotic stewardship: themes, subthemes, categories and exemplar quotations

Subthemes	Categories	Quotations
Familiarity with terms	Empirical therapy was perceived as rational use	***R2:*** *Proper use or rational use here is, if we suspect infection we start antibiotic even if we are not sure the infection is bacterial or viral. It is a tertiary care hospital so some time we do perform microbiology tests but not commonly. We suspect we prescribe the antibiotic which can work for everything.* ***R11****: Basically our hospital is teaching hospital we give antibiotic on proper time and with proper dose. We never give antibiotic without its need. Sometimes we do when have to give it prophylactically; patient is admitted and has exposure with infections. Here antibiotics are used rationally.*
	Irrational use of antibiotics	***R13:*** *We give medicine on patient demand it happens. We don’t go for cultures and blindly give triple therapy or a broad spectrum antibiotic.*
	Only few were familiar with the term “antibiotic stewardship” Majority were not familiar with the term “antibiotic time-out”	***R3****: It is multidisciplinary programme, consist first starting from of physicians, pathologist/microbiologist and pharmacologist. They work together on incidence and prevalence of infection and on the basis of it form an antibiogram and then proper empirical treatment is decided and proper use of antibiotics takes place.* ***R4:*** *I don’t know anything about that programme. And that kind of system is not practiced here.* ***R15:*** *I don’t know.it is very difficult word? What does it mean? And nothing like this is practiced here or known about.*

### Theme 2: antibiotic prescription practices

Broad spectrum antibiotics were considered as better treatment option by the doctors (8 out of 17 respondents). Moreover, broad spectrum antibiotics were prescribed as prophylactic agents to patients for nosocomial infections. Patient’s poor financial status and limited resources in public sector hospitals were highlighted by nine doctors as main reasons why culture tests were not performed. Unreliable laboratory reports were highlighted (2 out of 17 respondents) as a reason for not sending specimens for cultures (Table 3).

**Table 3:** Antibiotic prescription practices: theme, subthemes, categories and exemplar quotations

Subthemes	Categories	***Quotations***
Use of broad spectrum antibiotics	• Provides good cover • Prophylactic use	***R3:*** *When you don’t know which microorganisms it is, you fire all the guns and it’s safe.* ***R4:*** *We give broad spectrum to make sure that the patient does not acquire any hospital acquired infection.*
Why bacteriology is not performed?	Patient perspective • Affordability	***R1:*** *We live in a country with limited resources. We know that patient has, for example, respiratory or urinary tract infection, there might be known organisms and these antibiotics are effective so we will not tease a person with limited financial resources.*
	Hospitals perspective • Limited resources • Diagnosis based on clinical judgement • Lack of inter department communication • Unreliable laboratory test results	***R4:*** *Everything is not diagnosed on base of culture sensitivity tests there are many clinical signs* e.g. *pneumonia, GI problem we start the treatment and if problem is not solved we go for further investigations and we have limited resources so we cannot send cultures for every patient.* ***R10:*** *Our bad scenario is that our labs are not up to date, you can conduct the same test from 3 different laboratories and all of the results will be different and culture tests reports take more than10 days so it is very time consuming.*
Laboratory investigations performed	Before prescribing antibiotics • Only complete blood count as base line investigation • Culture tests were uncommon	***R1:*** *In government setup, we carry out basic investigations which indicate patient is having infection but what sort of infection what sort of organism he or she is having we cannot say anything about it.* ***R11:*** *No trend of culture tests. We only do it if condition of patient is worse and no medicine is working against it.*
	Investigations adopted to check ‘antibiotic timeout’ • Improvement in apparent condition • Check leucocyte count • No specific investigations for ‘antibiotic timeout’	***R5:*** *Mainly improvement of symptoms, and then urine culture, blood tests showing improvement in total leucocyte count shows medicine is effective.* ***R3:*** *In severe cases we have to give empiric treatment and it is recommended but after 72 h if you are asking about whether we check responses, we usually don’t.*

### Theme 3: antibiotic resistance

Most of the respondents (15 out of 17) stated that situation of AR is alarming in Pakistan. The doctors highlighted frequent use of antibiotics for minor ailments (9 out of 17 respondents) and as one of the main reasons of AR. Six out of 17 respondents stated that poor compliance and improper dosing contribute to already existing problem of antibiotic resistance. Lack of doctors’ experience (2 out of 17) and quacks working as doctors in remote areas of Pakistan (8 out of 17 respondents) were also reported as contributing factors that aggravated the problem of resistance. Four out of 17 doctors described that antimicrobial resistance leads to increase in out-of-pocket expenses of patients (Table 4).

**Table 4:** Antibiotic resistance: themes, subthemes, categories and exemplar quotations

Subthemes	Categories	Quotations
Alarming situation	Escalating resistance against various infectious disease	***R3:*** *Drug resistance is the major problem. In quinolone here drug resistance is up to 48% and this is very alarming. While sitting in South East Punjab, even in tuberculosis resistance has increased a lot.* ***R4****: I am working in neonatology unit most of the neonates are resistance to most of the broad spectrum antibiotics. if we see culture reports you can see that bacteria are resistant to 90% drugs.*
Reasons of resistance	Frequent use of antibiotics for minor ailments	***R2:*** *There is no need of antibiotics in viral infection like flu, and viral diarrhea in children, but antibiotics are used and prescribed for such ailments.*
	Demand of antibiotic by patient for minor ailments	***R4****:Doctor just give them painkiller and then they ask why you have not given us antibiotic.*
	Improper dosing and poor compliance	***R6:*** *Patient does not take medicine in exact dose and for complete duration. Patients take it for 2 or 3 days and discontinue because of many reasons.*
	Lack of qualified professionals	***R8:*** *I worked in primary healthcare center. There antibiotics are used irrationally specifically in children by quacks, who are non-qualified and not licensed to prescribe.*
	Limited number of antibiotics in public healthcare sector	***R3:*** *In quinolone if we require ciprofloxacin we sometimes get moxifloxacin. And in the same way we do not get the required dosage form. Dose cannot be adjustment. We have not many options available.*
	Lack of experience drives inappropriate use	***R3:*** *There is no guidance for health care professionals. Junior doctors (having little or no experience) use broad spectrum antibiotics for minor infections.*
Challenges associated with antibiotic resistance	Threat to effective antibiotic options for treatment Expensive drugs are the only option	***R7:*** *In next 10 years more than 60% antibiotics will become resistant and useless. After some years it may be impossible to treat patients with antibiotics that are available now.* ***R9:*** *New antibiotics are costly and new generations are not easily available.*
	Effect on patient • Recurrent infection • Financial burden on patient	***R1****: Patients suffer financially and ends up having recurrent infection.* ***R3:*** *Patient remains untreated because we have limited options. Patients cannot afford to buy purchase new antibiotics from market and they end up having recurrent infection.*

Table 5 describes the most commonly known resistant bacterial species reported by the study participants. The study participants stated that, mostly, gram negative bacterial species were resistant to commonly used antibiotics.

**Table 5:** Organisms for which drug resistance was commonly reported

Resistant organisms	
Gram negative bacteria	*Salmonella spp*
	*Pseudomonas aeruginosa*
	*Escherichia coli*
	*Klebsiella pneumoniae*
	*Stenotrophomonas maltophilia*
	*Acinetobacter spp*
	*Haemophilus influenza*
	*Morganella morganii*
Gram positive bacteria	*MRSA (methicillin resistant Staphylococcus aureus)*
Acid fast bacteria	*Mycobacterium tuberculosis*

### Theme 4: limited strategies adopted by the hospital administration to ensure quality and safe distribution of antibiotics

Most of the respondents agreed that there is no proper system to ensure the safe use of antibiotics (15 out of 17 respondents). They agreed that, although, policies and rules exist to ensure the quality and safe distribution of antibiotics but they are usually not followed. However, some study participants pointed out different strategies that were adopted to ensure safe use of antibiotics. Two out of 17 respondents stated that if a medicine was showing some adverse drug event, it was reported to hospital pharmacist. One respondent stated that pharmaceutical products containing antibiotics were sent to drug testing laboratory to check whether these meet quality standards. One respondent stated that one positive strategy to ensure rational use of antibiotics is to prescribe antibiotics from hospital essential list (Table 6).

**Table 6:** Limited strategies adopted by hospital administration to ensure quality and safe distribution of antibiotics: themes, subthemes, categories and exemplar quotations

Subthemes	Categories	Quotations
Reporting system	Adverse drug event reporting to hospital pharmacist is minimal	***R1:****Drug is showing serious side effect, dosage form is not proper, or drug is substandard we report it to pharmacist but it rarely happens that we report as there is no proper reporting system.*
	If medicine is substandard • Report to company • Obligatory drug testing to ensure the quality	***R5:*** *There is protocol called recall medicine protocol. We fill the form and report it to company. And ideally district drug controller should be informed.* ***R2****: Drug testing laboratory for testing quality of antibiotics if we see any problem like even given in proper dose and medicine is not effective.*

### Theme 5: implementation of antibiotic stewardship programme: barriers, suggestions and future benefits

Multiple reasons were highlighted by the respondents that were considered as barriers for successful implementation of antibiotic stewardship programme. Fifteen out of 17 respondents stated that no educational programme or awareness campaign existed to educate doctors about antibiotic stewardship. Lack of hospital antibiogram was considered as a major barrier which hindered the implementation of antibiotic stewardship program (10 out of 17 respondents). The respondents suggested that the government support and strict legislation regarding use and dispensing of antibiotics was important to ensure safe use of antibiotics (4 out of 17 respondents). More than half (9 out of 17 respondents) of the respondents stated that general public should be made aware to avoid self-medication of antibiotics, and they must also avoid visiting quacks (Table 7).

**Table 7:** Implementation of antibiotic stewardship programme: Barriers, suggestions and future benefits: themes, subthemes, categories and exemplar quotations

Subthemes	Categories	***Quotations***
Barriers to successful implementation	Workshops on antibiotic stewardship were not conducted	***R3:*** *There are very few people in this setting who talk about this or know about this. But no one owns it and its importance is neglected. No awareness workshop has been conducted regarding this programme in this setting.*
	Lack of proper audit system	***R5:*** *Accessibility to antibiotics is very easy that everybody can prescribe it like dispenser, junior doctors, trainee doctors,* etc.
	Lack of updated knowledge for qualified professionals	***R8:*** *Doctors are prescribing medicines that are not necessary and even those which are no more used in many other countries and are not necessary and no guidance for health care professionals is available.*
	Unavailability of antibiotic use guidelines and hospital antibiogram	***R9:*** *The hospital did not provide us antibiotic use guidelines.*
		***R15****: There is no antibiogram available in hospital to guide us about resistant organisms and treatment options.*
		***R3:*** *Our hospital has no specific guidelines. For bronchitis we follow British Thoracic Society guidelines.*
Suggestions	Strict enforcement of ongoing and new legislations	***R4:*** *We can implement this programme by bringing policies and it will require a lot of effort and home work. We should also make our public and physicians aware that antibiotics are not those medicines which you should use causally.*
	Active participation of health care professionals	***R1****: General practitioner should be made aware about rational use because patients visit them first. Pharmacist and doctor should collaborate and work together to ensure safe use of antibiotics.*
	Awareness among general public about antibiotic resistance and proper use	***R5:*** *We should educate our public that they should avoid self-medication of antibiotics and should not take it from unqualified professional.*
Future benefits of antibiotic stewardship programme	Might help in current issue of antibiotic resistance Helps in rational prescribing	***R4:*** *Right medicine will be given to patient.* ***R7:*** *It will educate doctors about latest guidelines and about resistance of antibiotics and by utilizing that awareness we can save our people from further resistance.*

## Discussion

Antimicrobial resistance is an issue that has been increasing globally with an alarming rate making it a huge concern [47]. Many interventions have been introduced to reduce incidence of AR [48, 49]. Such interventions are considered essential part of healthcare settings; one of such is ASP. This is one of the few studies conducted in Pakistan to access the knowledge of doctors about ASP, besides, it also explored the prescription practices of doctors and their views on situation of AR and its contributing factors. The perception of doctors about rational use of antibiotics and ASP was poor. Published papers have shown that physicians rarely improve their prescribing practices unless they have good knowledge and perception about antibiotic use and ASP [50].

Findings of this study revealed that the doctors used to prescribe broad spectrum antibiotics as an empiric therapy. Our participants also agreed that habitual empiric prescribing practices lead to inappropriate use of antibiotics which subsequently lead to AR. These results were similar to studies conducted in Cambodia and Iran that described the similar kind of behavior [51, 52]. Earlier study described that the inappropriate use of antibiotics may lead to prolonged hospital stay, increased cost of treatment, need for additional antimicrobial therapy, adverse patient outcomes and development of AR [53, 54]. No doubt, selection of antibiotics should be based on the culture and sensitivity results. However, delays in the availability of microbiology reports, lack of trust of doctors on the laboratory reports, burden on the laboratories of public sector hospitals, limited availability of antibiotics in the hospitals and poor financial status of patients compel the doctors to prescribe broad spectrum antibiotics. The same reasons of inappropriate use of antibiotics were highlighted in our study and a study from India [55].

The present study also revealed that the doctors used to prescribe antibiotics in almost all hospitalized patients as a prophylactic measure to counter the nosocomial infections. Similarly, a study from India also revealed that doctors prescribed antibiotics in hospitalized patients to avoid superinfections [56]. Findings of this study also revealed the patients also influenced doctors’ prescription by compelling them to include an antibiotic in their prescription to achieve fastest cure. This finding was similar to the studies from Sri Lanka and the UK which explained that the patients influenced that doctors to prescribe antibiotics, even when these were not required [57, 58].

A multicounty study conducted in European countries revealed that antibiotic guidelines help doctors in rational prescribing of antibiotics [59]. However, our study findings revealed that antibiotic use guidelines were not available in the hospitals, and the doctors used to prescribe antibiotics based on their clinical experience. The same antibiotic prescribing practices were reported in studies from India and Iran [52, 55], while, a study conducted in Saudi Arabia revealed to have antibiotics use guidelines in hospitals [60]. It is a good omen that our study participants were aware of the importance of having guidelines and felt that antibiotic prescribing practices could be improved upon availability of guidelines and information on local AR patterns. Likewise in a study from Ireland, doctors reported that the availability of local AR patterns could improve their antibiotic prescribing practices and contribute to improved patient outcomes [61]. Hospital antibiogram helps the doctors to select most effective antibiotic for the patients. However, according to our study participants, hospital antibiogram was not available in their hospitals. A study from the US showed improvements in antibiotic use if an antibiogram existed in a hospital [62]. Findings of this study also revealed that no audits were conducted in their hospitals to check the alarming rise in antibiotic use and AR. While, a study conducted in Kuwait showed audits helped in rational prescribing and prescription of antibiotics according to guidelines [63]. Availability of institutional antibiogram, antibiotic prescribing guidelines, educational sessions, advice from an antimicrobial stewardship team, regular audits and feedback strategies can greatly improve the rational antibiotic prescribing and the same was emphasized in earlier studies from Pakistan [30, 31]. According to a review, online training and mentoring programmes, national guidelines, and social media in stewardship all contributed to the success of antimicrobial stewardship programmes in the UK, the US, South Africa, Australia, and Colombia [17]. Likewise, the effectiveness of antibiotic stewardship interventions was evidenced by a review based on studies from LMICs, which found that all types of antibiotic stewardship interventions improved antibiotic prescribing and clinical outcomes [18]. Despite the limited but well-established benefits of ASP, another review indicated that LMICs face resource constraints, making it difficult for them to adopt “a WHO Practical ASP toolkit” in order to develop and sustain ASPs [19].

This study revealed that doctors had no idea about ASP, and it was relatively a new word for them. These findings were similar to an Indian study [55], while a Namibian study showed high awareness about ASP among healthcare professionals, although, it was not implemented with all its core elements [64]. Lack of our participants’ knowledge about ASP was either due to lack of training opportunities for them or due to the fact that they did not have any compulsion to attend such seminars. The interviewees also considered that these seminars did not benefit their professional career. Unfortunately, in Pakistan, there is no credit point system that could be added to profile of healthcare professionals. According to the WHO, training on antimicrobial stewardship improve the competency of healthcare professionals for judicious use of antibiotics. For this, the WHO offers courses for the clinicians, for example, “antimicrobial stewardship – a competency-based approach” [65].

This study has few limitations as well. First, a convenient sampling method was used to collect data. In convenience samples, subjects who are more easily accessible to the researcher are more likely to be included and, therefore, not all qualified individuals in the target population have the same opportunity to participate and study results are not much reliable and necessarily generalizable to this population. Though, increasing the sample size in quantitative studies can improve the credibility of convenience samples, but this is difficult in qualitative studies because saturation point criterion is used as redundancy signal. However, considering aforementioned concerns, a mix of purposive and convenience sampling was adopted and we believe that the utilization of purposive sampling in the first phase surpassed any possible concerns associated with convenient sampling and saturation point criteria altogether. Second, our findings cannot be generalized to the whole of Pakistan as these depict the views of doctors working in two main cities of southern Punjab. However, the authors do not expect significant differences across the country, as the same healthcare system has been implemented and regulated throughout the country by the Ministry of National Health Services, Regulation and Coordination. Though this study provides an indication of what is happening in the whole province of Punjab, but there is a need for large scale multi-setting quantitative studies to underline more reliable and impactful country-level situation. Likewise, multi-perspective studies involving regulators, pharmacists, nurses and dispensers are encouraged to improve the antibiotic stewardship activities at each level of healthcare through evidence based recommendations.

### Implication of findings on policy and practice

The strength of the present study is that it highlighted an important issue about which limited data is available from developing countries, for example, Pakistan. In terms of policy, our study findings warrant the need to put legislation to introduce the ASP in hospital settings in Pakistan. Moreover, National Action Plan of Pakistan on AR must be implemented to prohibit irrational use of antibiotics and to implement ASP. In practice terms, there is a dire need regarding training and involvement of doctors in ASP to minimize the surge in the antimicrobial resistance issue. Frequent seminars must be conducted on small and large scale to educate doctors about the benefits of implementing ASP, and warn them to prescribe antibiotics cautiously because of the fact that our study participants imperfectly understood the concept of empirical therapy and ASP. Yet education alone will not be enough, antibiotic prescribing guidelines and local antibiogram must be available in each hospital. In addition, collaborative efforts by all the healthcare professionals dealing with human as well as animal patients are needed to adopt “One Health Approach” with the aim to halt the spread of antibiotic resistance at each level. Future large scale interventional studies involving doctors, pharmacists, nurses, veterinary and agricultural experts and health policy makers are also required to assess the impact of ASP on health outcomes and to understand the potential barriers and facilitators for implementation of program.

The study findings will not only aid countries with existing ASPs in forthcoming initiatives, but also those countries that are planning to implement ASPs can gain insight for effective introduction of programme. Specifically, the findings of this study highlight that countries with existing ASPs should focus on awareness and education of healthcare professionals about ASPs protocols, strict compliance with the antibiotic prescribing guidelines, active participation of healthcare professionals, and availability of local antibiogram in each healthcare facility. Whereas, the countries planning to implement ASPs can learn that special attention to the above mentioned aspects is mandatory for an effective introduction of activities. Moreover, the study findings will act as a strong base and impetus for both large scale multi-stakeholder qualitative studies and quantitative studies.

## Conclusion

The study concluded that the doctors working in the public sector hospitals of Pakistan had inadequate knowledge about ASP. There was no proper system in place to ensure the appropriate use of antibiotics. Similarly, local antibiogram data and antibiotic prescribing guidelines were not available in the hospital. There is dire need to implement the strategic objectives of national action plan of Pakistan on AMR in order to mitigate the adverse outcomes of AR in future.

## Supplementary Information

**Additional file 1.** Interview schema

## Data Availability

The raw data will be provided upon receiving reasonable request. Please contact corresponding author at pharmacist_atif@yahoo.com

## Abbreviations

ASP: Antibiotic Stewardship Programs
LMICs: Low- and Middle-Income Countries
DDD: Defined Daily Dose
MDR: Multidrug Resistant
CDC: Centers for Disease Control And Prevention
AR: Antibiotic Resistance
IPC: Infection Prevention and Control
GAP: Global Action Plan
BVH: Bahawal Victoria Hospital
COREQ: Consolidated Criteria for Reporting Qualitative Studies
