# Supplementary material for: Antibiotic stewardship program in Pakistan: a multicenter qualitative study exploring medical doctors’ knowledge, perception and practices
Source: BMC Infect Dis. 2021 Apr 21;21:374. doi: 10.1186/s12879-021-06043-5 (PMC8059254; doi:10.1186/s12879-021-06043-5)
Supplement: Supplementary file 1 — Additional file 1. Interview schema [file 12879_2021_6043_MOESM1_ESM.docx]

**Additional File 1**

**Interview schema**

|  | **Discussion Topics** | **Probes** |
| --- | --- | --- |
|  | **General information**  Gender __________________________________________________________________  What is your primary work area or unit in this institution? _________________________  What is your staff position in this institution? ___________________________________  Since how long you have been working in this institution? _________________________  Since how long you have been working in your current specialty? ________________ | |
| **1** | What do you know about the rational use of antibiotics in hospital? |  |
| **2** | Do you think inappropriate use of antibiotics is the problem in Pakistan/in this hospital? | - If yes, then please explain how healthcare system and patient suffer. |
| **3** | Why do prescribers tend to prescribe broad-spectrum antibiotics more commonly? | - How do you compare the use of broad vs. narrow spectrum antibiotics? |
| **5** | How frequently do you encounter drug resistant organisms within your clinical practice? | - Which types of pathogens/infections do you think have more resistance profile? |
| **7** | Do you face any challenge in your daily practice as a result of antimicrobial resistance? | - How does it affect the patient? |
|  | How do you describe the current status of antimicrobial resistance? | - At institutional and national level |
| **8** | What audit methodology is used to monitor the quality of antibiotic prescriptions at your facility? |  |
| **9** | What type of investigation procedures are adopted in your setting to diagnose the causative agent before starting treatment? | - If antibiotics are prescribed without any investigations (apart from emergency) what is the reason for this? |
| **11** | What are the formal procedures used to check the appropriateness of antibiotics after initial 48 hours of order? (Antibiotics timeout) | - Is any antibiotic prescription/use guideline available in your hospital? |
| **14** | What do you know about antibiotic stewardship program? |  |
| **15** | What types of workshops or awareness programs are arranged in your setting to educate doctors about this program? | - What do you learned from it? - How such awareness seminar/conferences effect the attitude of prescribers towards prescribing? |
| **16** | What do you think is the future of this program in Pakistan and particularly in this institution? |  |
| **17** | What could we do to implement this program in this facility and how it would benefit this facility? |  |
